# Utility of the simplified measurements of muscle mass in patients with gastrointestinal and chronic liver diseases

**DOI:** 10.1038/s41598-020-67847-0

**Published:** 2020-07-01

**Authors:** Hitomi Takada, Fumitake Amemiya, Tomoki Yasumura, Hiroki Yoda, Tetsuya Okuwaki, Keisuke Tanaka, Makoto Kadokura, Nobuyuki Enomoto

**Affiliations:** 1 Department of Gastroenterology and Hepatology, Municipal Hospital of Kofu, 366 Masutsubo-cho, Kofu-city, Yamanashi 400-0832 Japan; 2 First Department of Internal Medicine, Faculty of Medicine, University of Yamanashi, Yamanashi, Japan

**Keywords:** Liver cirrhosis, Risk factors

## Abstract

Sarcopenia is an important prognostic factor in patients with gastrointestinal and chronic liver diseases. Computed tomography and bioelectrical impedance analysis are the gold standards for measuring skeletal muscle mass for the diagnosis of decreased muscle mass, but there are some institutions where BIA and CT cannot be carried out. We evaluated the utility of simplified methods for measuring muscle mass; the psoas muscle mass index (PMI) method, simple PMI method, and arm muscle area (AMA) method. This retrospective study included 331 patients with gastrointestinal diseases and 81 patients with chronic liver diseases who were admitted from June 2018 to December 2019 at Municipal Hospital of Kofu. The skeletal muscle mass was measured using the PMI via the volume analyzer SYNAPSE VINCENT ver3.0, simple PMI based on CT imaging, and AMA method. Positive correlations were found between muscle mass measured by PMI and simple PMI, PMI and AMA, and simple PMI and AMA in patients with gastrointestinal diseases (correlation coefficients = 0.76, 0.57, 0.47, respectively, p < 0.001). Positive correlations were observed between muscle mass measured by PMI and simple PMI, PMI and AMA, and simple PMI and AMA in chronic liver diseases (correlation coefficients = 0.77, 0.53, 0.45, respectively, p < 0.001). Measurement of muscle mass by the AMA method showed some correlation with the PMI method. Measurement of muscle mass by the simple PMI method showed correlation with the PMI method. These simplified methods can be alternative methods of evaluating muscle mass in patients with gastrointestinal and chronic liver disease.

## Introduction

Sarcopenia is a syndrome characterized by a decrease in skeletal muscle mass, skeletal muscle strength, and physical function^1^. There are several diagnostic criteria for sarcopenia, including the European Working Group on Sarcopenia in Older People (EWGSOP), the International Working Group on Sarcopenia (IWGS), the Asian Working Group for Sarcopenia (AWGS) criteria, and the Japanese Society of Hepatology (JSH)^2–7^. All definitions are based on decreased skeletal muscle mass and decreased function, and decreased skeletal muscle mass is defined as myopenia. In the JSH criteria, patients with chronic liver disease are diagnosed with sarcopenia if they have “decreased grip strength” and “decreased muscle mass” (as determined by computed tomography (CT) or bioelectrical impedance analysis (BIA)-guided skeletal muscle mass index). CT and BIA are the gold standards for measuring skeletal muscle mass available at present. Performing these examinations is difficult in some institutions with no special software or equipment. Simplified methods for measuring muscle mass have attracted attention in such clinical settings.

One of the simplified methods is the psoas muscle mass index (PMI) using CT images. This method is mentioned as an alternative method in the criteria from the JSH^7^. In particular, simple PMI can be obtained from CT images immediately and easily. The second method uses anthropometric measurements to estimate the area of the brachial muscle. Arm circumference (AC) and triceps skinfold thickness (TSF) are used to estimate muscle mass by calculating the arm muscle area (AMA) of the brachial muscle^8,9^. In this study, the utility of the AMA method and the simple PMI method as simplified methods was verified.

## Methods

### Patients

This study targets 331 patients with gastrointestinal diseases who were admitted to our department between June 2018 and December 2019 and whose muscle mass was measured using three methods: the psoas muscle mass index (PMI) method, the simple PMI method, and the arm muscle area (AMA) method. The patients with comorbidity malignancies other than gastrointestinal cancers were excluded. All patients provided informed consent for this study, which was in compliance with the Declaration of Helsinki and was approved by the ethics committee for clinical studies of Municipal Hospital of Kofu: Rinshoukenkyu-Rinrishinsa-Iinkai (in Japanese), approval number 31–2).

### Measurement of muscle mass and diagnosis using the PMI method and simple PMI method

CT images taken during hospitalization or within one month before admission were used. SYNAPSE Vincent volume analyzer version 3.0 was used in the PMI method as the sum of the areas of the iliopsoas muscles on both sides at the level of the L3 vertebral body divided by the square of the height. The simple PMI was obtained as the sum of the product of the long axis and the short axis of the iliopsoas muscles on both sides at the level of the L3 vertebral body on CT divided by the square of the height. According to the diagnostic criteria for sarcopenia in patients with liver disease from the JSH, the cut-off value for myopenia was a PMI of 6.36 cm^2^/m^2^ in males and 3.92 cm^2^/m^2^ in females, and a simple PMI of 6.0 cm^2^/m^2^ in males and 3.4 cm^2^/m^2^ in females^7^.

### Measurement of muscle mass and diagnosis using the AMA method

Measurements were performed on the non-paralytic or non-dominant upper arm using an insert tape and an adipometer (ABBOTT JAPAN). Arm circumference (AC) and triceps skinfold thickness (TSF) were measured at the level of the midpoint between the acromial process of the scapula and the olecranon process of the ulna. All measurements were performed three times, and mean values were used. Arm muscle circumference (AMC) and AMA were calculated using the following equations. There are no standard values recommended as cut-offs for myopenia, and 21.4 cm^2^ was used for males and 21.6 cm^2^ for females in this study as these are the cut-offs for undernutrition in the general elderly population^8,9^. The percentage notation was calculated with reference to the Japanese Anthropometric Reference Data (JARD2001).

$${\text{AMC}}\, = \,{\text{AC}} - \pi \times {\text{TSF}}.$$

AMA = AMC (cm)^2^/4π – bone area (males 10 cm^2^, females 6.5 cm^2^).

### Statistical analyses

Values were shown as means ± standard deviation (SD). Categorical variables were subjected to Fisher's test. Continuous variables were using unpaired Student’s t-tests. Pearson's product rate correlation was used to assess the correlation of continuous variables. The best cut-off values in receiver operating characteristic (ROC) analyses were determined by the Youden index. P value < 0.05 was considered statistically significant. All statistical analyses were performed using EZR (Saitama Medical Center, Jichi Medical University, Saitama, Japan), a graphical user interface for R (The R Foundation for Statistical Computing, Vienna, Austria). More precisely, it is a modified version of the R commander designed to include statistical functions frequently used in biostatistics.

## Results

### Background characteristics of gastrointestinal disease patients

The background characteristics of the 331 patients with gastrointestinal diseases who underwent muscle mass measurements are shown in Table 1. The primary diseases included liver cirrhosis in 81 patients [including 54 patients with hepatocellular carcinoma (HCC)], gastric or esophageal cancer in 34 patients, biliary or pancreatic cancer in 47 patients, colorectal cancer in 29 patients, and benign disease in 140 patients. Benign diseases included colorectal adenomas in 15 patients, gastrointestinal bleeding in 17 patients, enteritis and intestinal obstruction in 28 patients, choledocholithiasis in 38 patients, acute pancreatitis in 14 patients, and other benign diseases in 28 patients. The median age was 74 ± 13 years old, with 206 (62%) males. Myopenia in patients with gastrointestinal diseases was observed in 115 (35%) patients by PMI, 102 (31%) by simple PMI, and 123 (37%) by AMA. The frequency of myopenia was significantly higher in patients with malignant tumors than in those without (24 vs. 5.7% by PMI, p < 0.001, 37 vs. 25% by simple PMI, p = 0.017, 49 vs. 26% by AMA, p < 0.001). The frequency of myopenia did not differ by BMI, blood test findings, or presence of comobidities in this study.

**Table 1:** Backgrounds of patients with gastrointestinal diseases.

		**N = 331**
Age: years, mean ± SD		74 ± 13
Men: n (%)		206 (62%)
BMI, mean ± SD		22 ± 4.2
Performance status: n (%)	0/1/2/3/4	105/110/74/35/7 (32/33/22/11/3%)
%AC: %, mean ± SD		89 ± 13
%AMC: %, mean ± SD		95 ± 13
%AMA: %, mean ± SD		69 ± 26
Primary disease: n (%)	Chronic liver disease	81 (24%)
	Gastroesophageal cancer	34 (10%)
	Biliary pancreatic cancer	47 (14%)
	Colorectal cancer	29 (9%)
	Benign disease	140 (43%)
Comorbidities: n (%)	Heart disease	54 (17%)
	Chronic lung disease	24 (7.3%)
	Cerebrovascular disease	44 (13%)
	Chronic renal disease	17 (5.2%)
	Diabetes	68 (21%)
Myopenia: n (%)	By PMI method	115 (35%)
	By simple PMI method	102 (31%)
	By AMA method	123 (37%)

Continuous values are expressed as mean ± standard deviation.

*BMI* body mass index, *AC* Arm circumference, *AMC* Arm muscle circumference, *AMA* Arm muscle area, *PMI* Psoas muscle mass index.

### Comparisons between muscle mass measurement methods in patients with gastrointestinal diseases

**Figure 1:**
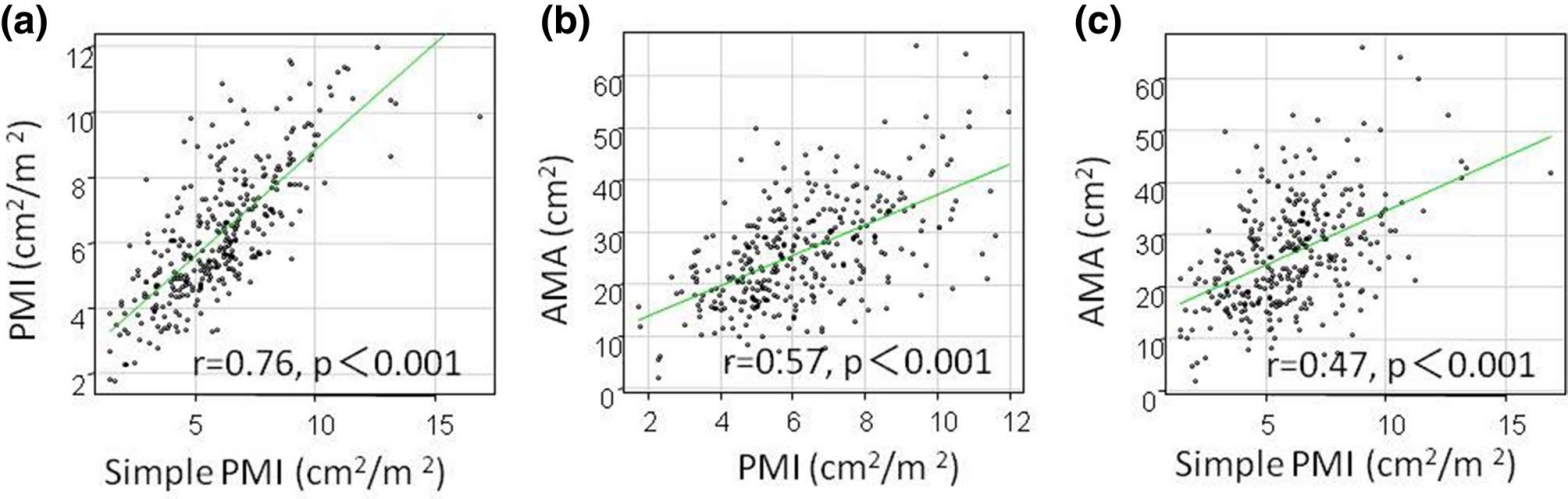
Comparison between muscle mass measurement methods in 331 patients with gastrointestinal diseases. (**a**) PMI and simple PMI, (**b**) PMI and AMA, (**c**) simple PMI and AMA.

**Figure 2:**
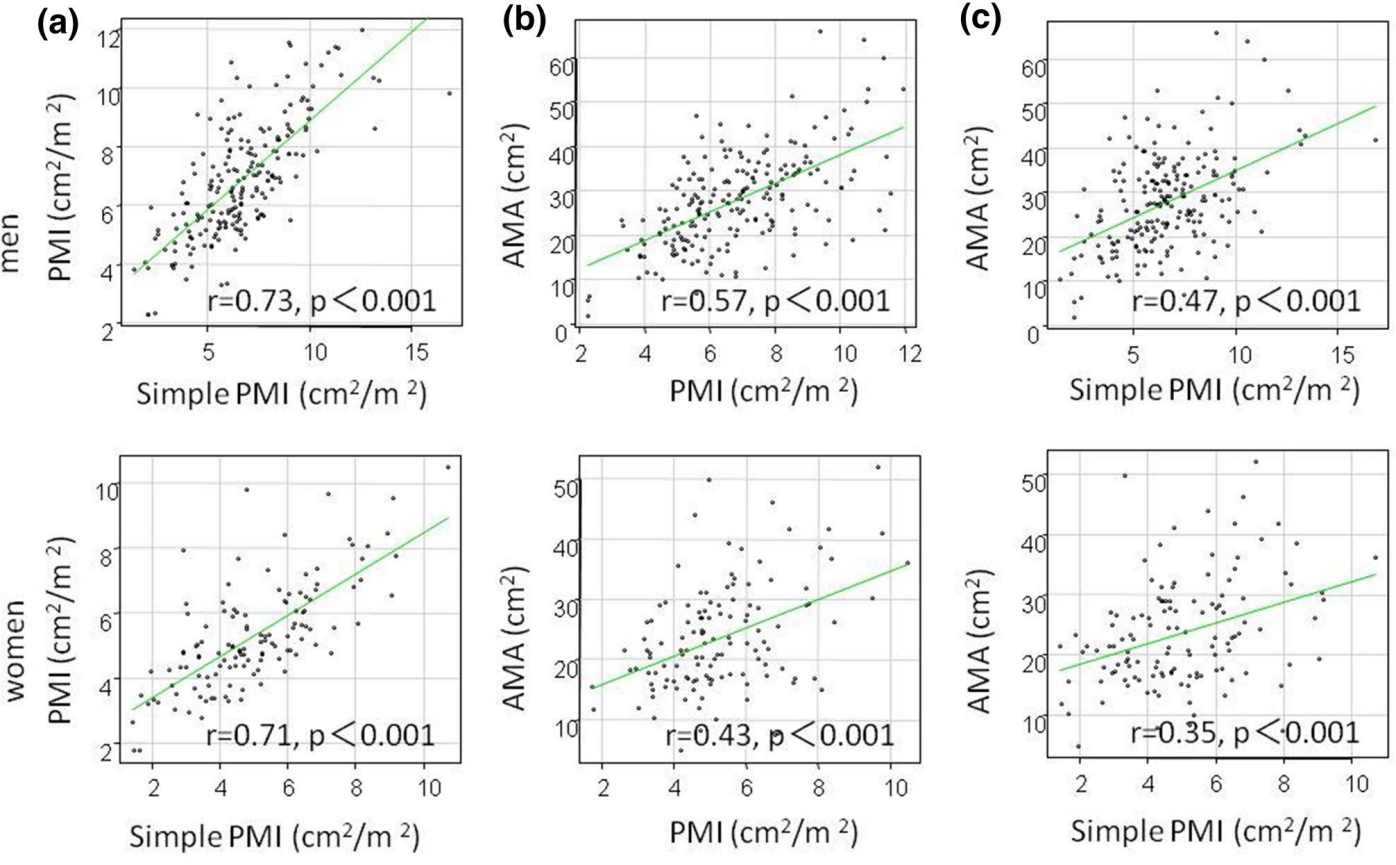
Comparison between muscle mass measurement methods by sex in patients with gastrointestinal diseases. (**a**) PMI and simple PMI, (**b**) PMI and AMA, (**c**) simple PMI and AMA.

Positive correlations were found between muscle mass measured by PMI and simple PMI, PMI and AMA, and simple PMI and AMA (correlation coefficients = 0.76, 0.57, 0.47, respectively, p < 0.001) (Fig. 1). Similarly, in males (correlation coefficients = 0.73, 0.59, 0.48, p < 0.001) and females (correlation coefficients = 0.71, 0.43, 0.35, p < 0.001), a positive correlation between the three methods was observed (Fig. 2).

### Background characteristics of chronic liver disease patients

The characteristics of chronic liver disease patients are shown in Table 2. All patients had liver cirrhosis, and 54 patients also had HCC. The median age was 75 ± 11 years old, with 54 (67%) males. Etiology of chronic liver disease was hepatitis B, hepatitis C, alcohol, non-alcoholic steatohepatitis, and others in 5, 39, 16, 19, and 2 patients. Hepatic function was Child–Pugh A, B, and C in 39, 32, and 10 patients. Myopenia was observed in patients with chronic liver disease in 29 (36%) patients by PMI, 23 (28%) by simple PMI, and 29 (36%) by AMA.

**Table 2:** Backgrounds of patients with chronic liver diseases.

		**N = 81**
Age: years, mean ± SD		75 ± 11
Men: n (%)		54 (67%)
BMI, mean ± SD		22 ± 3.7
Performance status: n (%)	0/1/2/3/4	19/31/21/8/2 (23/38/26/10/3%)
%AC: %, mean ± SD		90 ± 13
%AMC: %, mean ± SD		95 ± 14
%AMA: %, mean ± SD		70 ± 28
HCC: n (%)		54 (67%)
Etiology: n (%)	HBV	5 (6.6%)
	HCV	39 (48%)
	Alcohol	16 (20%)
	NASH	19 (23%)
	AIH	1 (1.2%)
	PBC	1 (1.2%)
Child–Pugh grade: n (%)	A/B/C	39/32/10 (48/40/2%)
Ascites: n (%)		18 (22%)
Comorbidities: n (%)	Heart disease	13 (16%)
	Chronic lung disease	8 (9.9%)
	Cerebrovascular disease	4 (4.9%)
	Chronic renal disease	6 (7.4%)
	Diabetes	23 (28%)
Oral medication: n (%)	BCAA	24 (30%)
	Diuretics	31 (38%)
	Carnitine	6 (7.4%)
Myopenia: n (%)	By PMI	29 (36%)
	By simple PMI	23 (28%)
	By AMA	29 (36%)
Albumin: g/dl, mean ± SD		3.4 ± 0.75
Total cholesterol: mg/dl, mean ± SD		179 ± 59
Total bilirubin: g/dl, mean ± SD		1.5 ± 1.5
γ-GTP: U/l, mean ± SD		140 ± 231
White blood cell: × 10^3^/μl, mean ± SD		5.9 ± 2.8
Hemoglobin: g/dl, mean ± SD		12 ± 2.2
Platelet: × 10^3^/μl, mean ± SD		157 ± 103
Neutrophil: %, mean ± SD		67 ± 13
Lymphophil: %, mean ± SD		23 ± 11
Prothrombin time: %, mean ± SD		80 ± 16

Continuous values are expressed as mean ± standard deviation.

*BMI* body mass index, *AC* Arm circumference, *AMC* Arm muscle circumference, *AMA* Arm muscle area, *HCC* hepatocellular carcinoma, *NASH* nonalcoholic steatohepatitis, *AIH* autoimmune hepatitis, *PBC* Primary biliary cholangitis, *BCAA* branched-chain amino acid, *PMI* Psoas muscle mass index, *γ-GTP* gamma- glutamyltransferase.

### Comparisons between muscle mass measurement methods in patients with chronic liver disease

**Figure 3:**
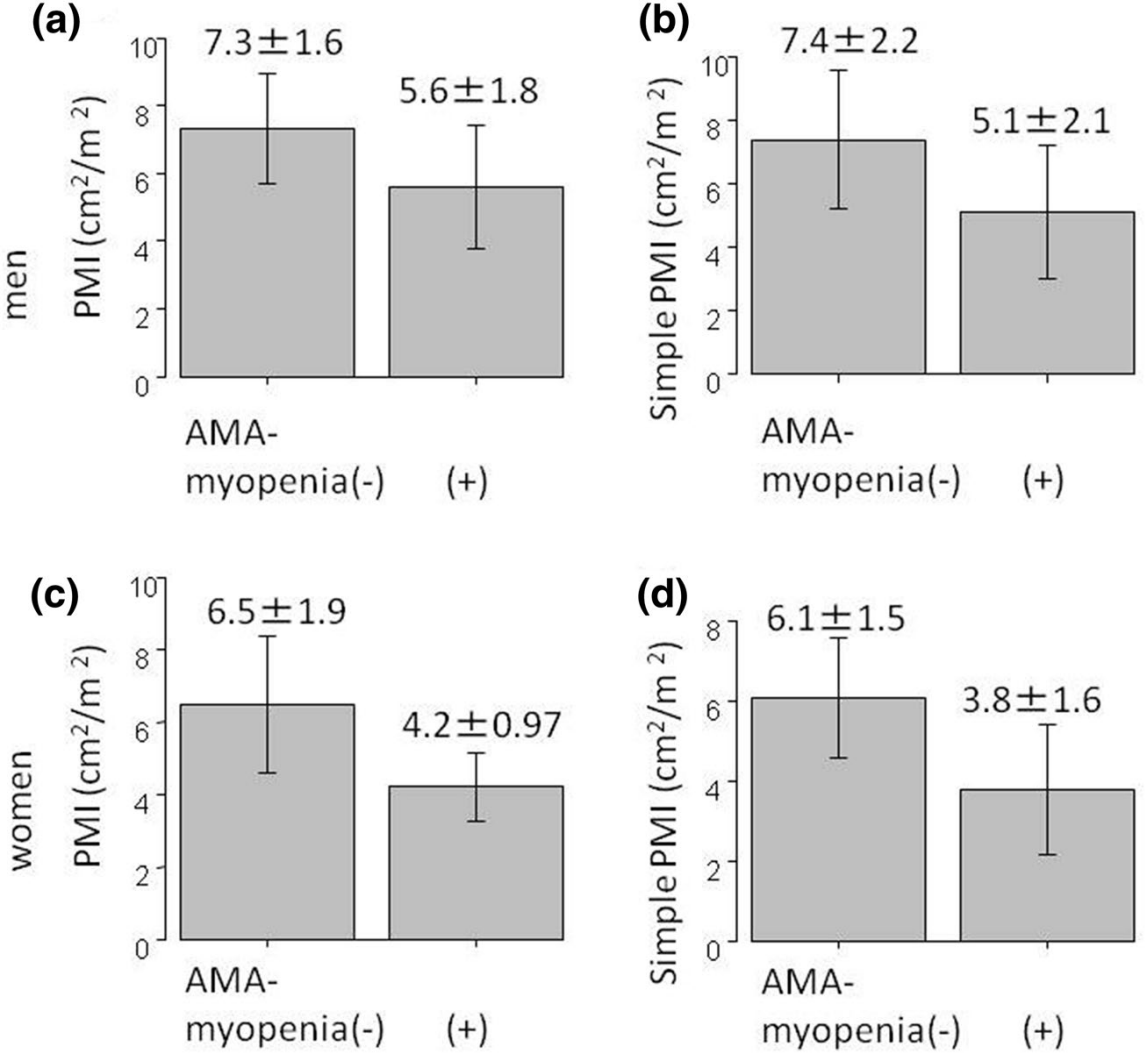
Muscle mass by the PMI/simple PMI method depending on the presence or absence of myopenia diagnosed by the AMA method in 81 patients with chronic liver diseases. (**a**) men and PMI, (**b**) men and simple PMI, (**c**) women and PMI, (**d**) women and simple PMI.

**Figure 4:**
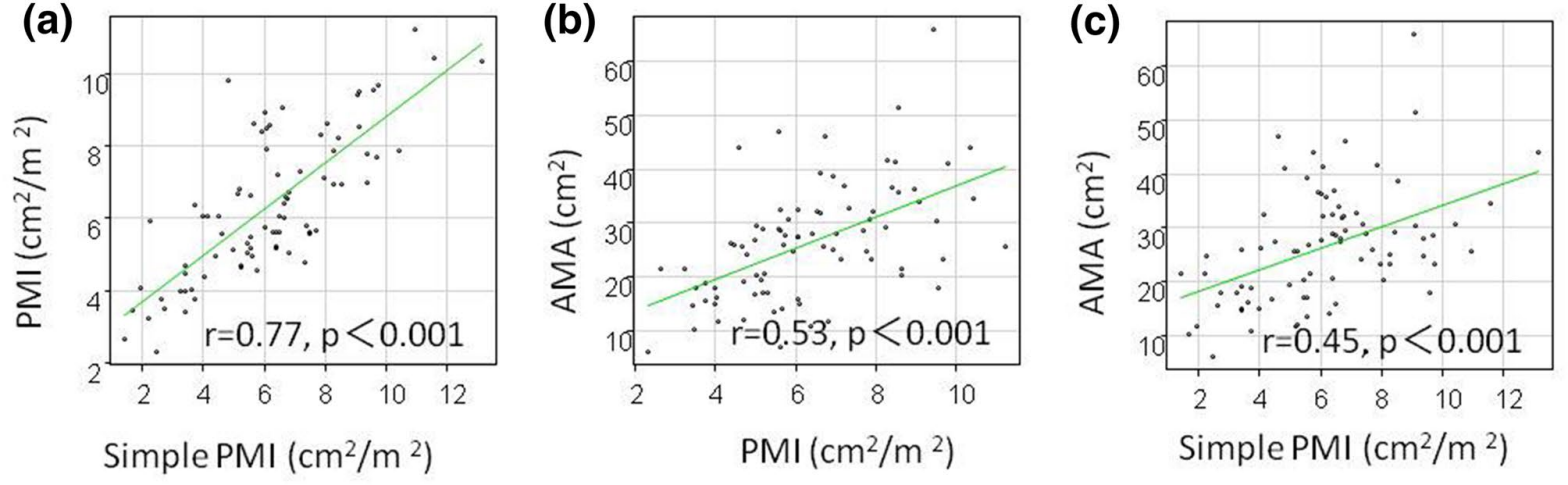
Comparison between muscle mass measurement methods in 81 patients with chronic liver diseases. (**a**) PMI and simple PMI, (**b**) PMI and AMA, (**c**) simple PMI and AMA.

**Figure 5:**
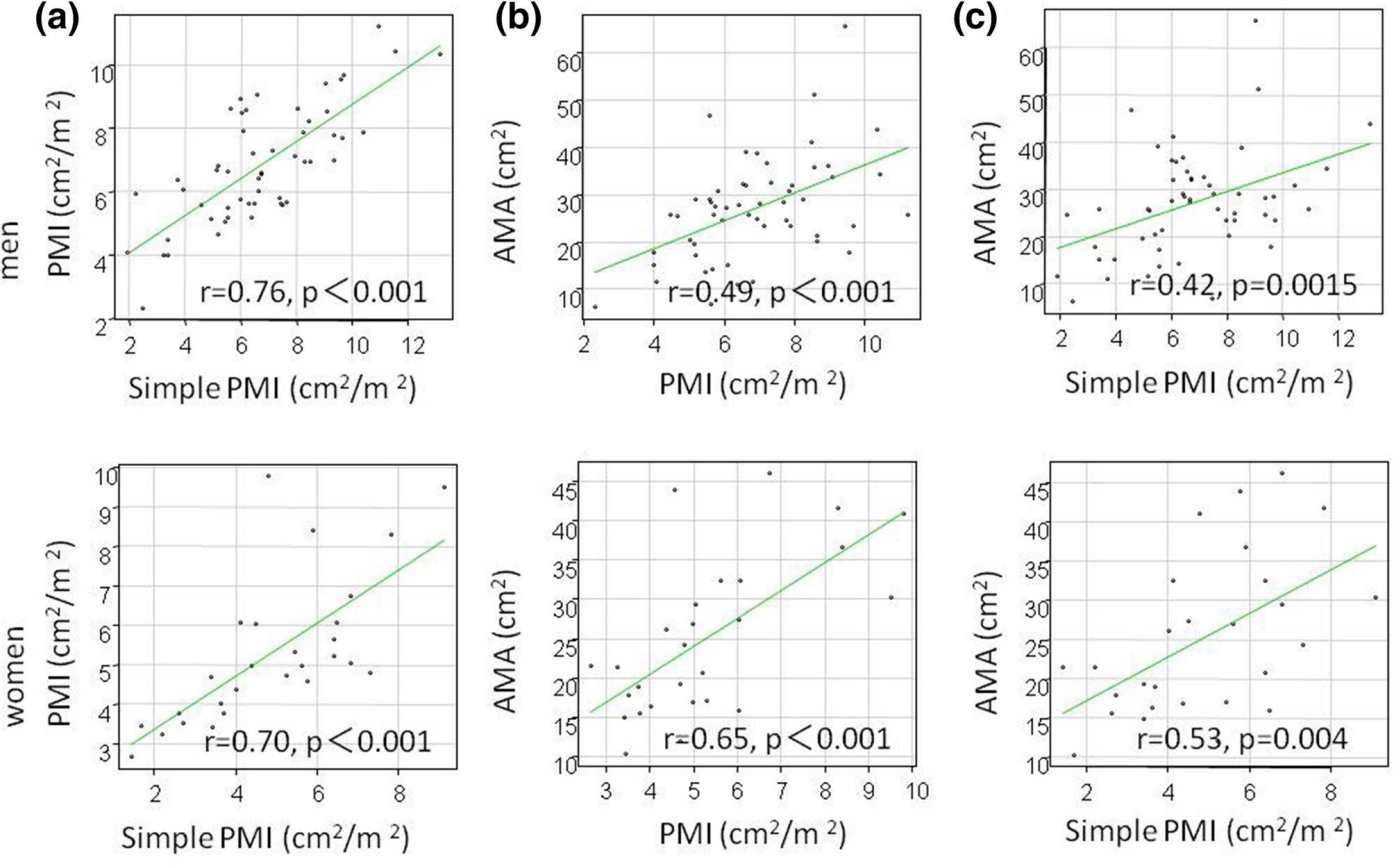
Comparison between muscle mass measurement methods by sex in patients with chronic liver diseases. (**a**) PMI and simple PMI, (**b**) PMI and AMA, (**c**) simple PMI and AMA.

Patients diagnosed with myopenia by the AMA method had significantly lower muscle mass by the PMI method compared to patients without myopenia (males 5.6 ± 1.8 vs. 7.3 ± 1 0.6 cm^2^/m^2^, p = 0.001, females 4.2 ± 0.97 vs. 6.5 ± 1.9 cm^2^/m^2^, p < 0.001) and the simple PMI method (males 5.1 ± 2.1 vs. 7.4 ± 2.2 cm^2^/m^2^, p = 0.001, females 3.8 ± 1.6 vs. 6.1 ± 1.5 cm^2^/m^2^, p < 0.001) (Fig. 3). Positive correlations were observed between muscle mass measured by PMI and simple PMI, PMI and AMA, and simple PMI and AMA (correlation coefficients = 0.77, 0.53, 0.45, respectively, p < 0.001) (Fig. 4). A positive correlation was also found among the three methods, in males (correlation coefficient = 0.76 p < 0.001, 0.49 p < 0.001, 0.42, p = 0.0015) and females (correlation coefficient = 0.70 p < 0.001, 0.65 p < 0.001, 0.53, p = 0.004), (Fig. 5).

### Accuracy in identifying myopenia using simple PMI and AMA in chronic liver disease patients

The reliability of simplified methods was as follows when the PMI method was assumed as the gold standard method: the sensitivity of the simple PMI method was 62% (95% CI 42–79%), specificity was 90% (95% CI 79–97%), the positive predictive value was 78% (95% CI 56–93%), and negative predictive value was 81% (95% CI 69–90%), while the sensitivity of the AMA method was 62% (95% CI 42–79%), specificity was 79% (95% CI 65–89%), the positive predictive value was 62% (95% CI 42–79%), and negative predictive value was 79% (95% CI 65–89%). The cut-off values for predicting PMI-induced myopenia were 21.4 cm^2^ (AUROC 0.74, 95% CI 0.61–0.88) in males and 21.4 cm^2^ (AUROC 0.81, 95% CI 0.65–0.98) in females, respectively.

## Discussion

Sarcopenia was proposed in 1989 by Rosenberg^1^ and, to date, there have been several diagnostic criteria^2–6,10^. Skeletal muscle mass index (SMI) measured by BIA and CT is the current standard for skeletal muscle mass, and sarcopenia is diagnosed in patients with chronic liver disease who have decreased grip strength (26 kg for males and 18 kg for females) and myopenia with SMI measured by CT (42 cm^2^/m^2^ in males and 38 cm^2^/m^2^ in females) or BIA (7.0 kg/m^2^ in males and 5.7 kg/m^2^ in females) in the JSH criteria^7^. In this study, we investigated the utility of simplified methods that did not require special equipment or software for measuring muscle mass. There was a positive correlation between muscle mass measured by the PMI method, simple PMI, and AMA method in patients with gastrointestinal diseases and chronic liver disease. In particular, the reliability of AMA method in diagnosing myopenia is not equal to PMI, but in institutions where CT cannot be done, we have thought that the AMA method was alternative method for evaluating muscle mass.

The PMI method is an evaluation method using CT imaging, and is easy to use in patients with HCC in whom CT scans are frequently performed in the follow-up. In the present study, PMI was calculated using the manual trace method proposed by Vincent ver3.0. PMI has been reported to correlate with SMI using muscle mass measuring software (r = 0.57, P < 0.01) and SMI measured by BIA (r = 0.74, P < 0.01)^11–13^. Additionally, simple PMI is one of the simplified methods referred to in the JSH criteria. A positive correlation between simple PMI and SMI has been reported (r = 0.57, p < 0.01)^7^.

The second simplified method of evaluating muscle mass is the AMA method; the method for estimating the cross-sectional area of the brachial muscle using anthropometric measurements. It has been reported to be strongly correlated with whole body skeletal muscle mass measured by dual energy X-ray absorptiometry and AMA in the elderly^8^. Moreover, there is reportedly a good correlation between AMA and grip strength in patients waiting for liver transplantation (Spearman correlation 0.49, p < 0.01)^14^. Among elderly individuals over 70 years and older, the mortality rate was reported to be higher in males with AMA ≤ 21.4 cm^2^ and ≤ 21.6 cm^2^ in females^9^. However, overestimation of the AMA method has been reported as 15–25% over actual muscle mass, and a large difference particularly in patients with thick subcutaneous fats was reported in the 1980–1990s^8^. A subsequent 2010 research reported that anthropometric AMA correlated well with CT-based AMA (r = 0.85, p < 0.001 in males and r = 0.90, p < 0.001 in females). Reproducibility and difficulty in establishing uniform cut-off values across races have been reported^15^. As a simplified anthropometric method other than the AMA method, a yubi-wakka test in Japanese patients has been reported; however, it is largely affected by leg edema and obesity^16–19^. In patients with chronic liver disease in whom edema of the lower legs is common, a determination based on measurements of the lower legs would likely be difficult. Thus, anthropometric AMA measurements that can be performed as a primary screening at any time and any place are helpful.

In this study, myopenia in patients with gastrointestinal diseases was found in 35% of patients with PMI, 31% with simple PMI, and 37% with AMA. Myopenia in patients with chronic liver disease was found in 36% of patients with PMI, 28% with simple PMI, and 36% with AMA. In a large survey of the general Japanese population, the incidence of sarcopenia was approximately 8% compared to 20% in individuals aged 65 years and older^20,21^. The prevalence of myopenia in the field of gastrointestinal cancers has been reported to be high at 26–65% for gastric and esophageal cancer^22–26^, 19–39% for colorectal cancer^27,28^, 21–63% for biliary and pancreatic cancer^29,30^, 11–65% for HCC^31–34^. In esophagectomy cases, postoperative cardiovascular-related complications, pulmonary complications, and mortality were significantly higher in patients with myopenia^22,23^. Sarcopenia in gastric cancer patients was associated with infection after surgery (odds ratio 9.0), independent factors for death within 1 year after surgery (hazard ratio 3.6), and factors related to long-term hospitalization^24,26^. In colorectal cancer patients treated with chemotherapy, the frequency of grade 3–4 toxicity was high in patients with sarcopenia, and the survival rate was significantly worse^27,28^. Myopenia in biliary and pancreatic cancer was an independent risk factor for survival and recurrence, independent of cancer progression^29,30^. Myopenia has been reported to be a prognostic factor independent of liver function in patients with compensated and early decompensated cirrhosis^31,35,36^. In patients with HCC, myopenia has been reported to correlate with prognoses in patients undergoing hepatectomy, liver transplantation, percutaneous radiofrequency ablation, or hepatic artery embolization. In particular, the association between myopenia and dose intensity in patients taking molecular-targeted drugs is of interest^32,37^. Screening myopenia is important for predicting prognoses and selecting treatments for patients with gastrointestinal diseases and chronic liver disease. The number of patients with chronic liver disease was small in our hospital, and sufficient statistical power was not obtained in this study. Assessment in more patients and detailed evaluations are necessary in the future.

Limitations of this study include its retrospective nature, the small sample size, and the lack of grip strength measurements. The significance of myopenia has also been reported to vary by sex^38–43^, but there were few female patients and sufficient evaluation by sex could not be carried out in this study. The relationship between myopenia and presence of comobidities and malignant tumors has not been adequately studied, and additional searching is needed.

Muscle mass measured by the AMA method and simple PMI method showed some correlation with muscle mass measured using the PMI method. In particular, the AMA method is a non-invasive muscle mass measurement method that can be performed without radiation exposure, and can be performed conveniently at any institution.

## Conclusion

Measurements of muscle mass by the AMA method and simple PMI method are correlated with measurement by the PMI method, and these methods can be simplified alternative methods of evaluating muscle mass in patients with gastrointestinal diseases and chronic liver disease.
